# MODIFIABLE DEMENTIA RISK FACTORS: THE EFFECTS OF BEHAVIOR MODIFICATION ON PERCEPTIONS OF AN OLDER ADULT AT RISK

**DOI:** 10.1093/geroni/igae098.4243

**Published:** 2024-12-31

**Authors:** Karli Poettcker, Molly Maxfield

**Affiliations:** Arizona State University, Phoenix, Arizona, United States; Arizona State University, Phoenix, Arizona, United States

## Abstract

Alzheimer’s disease and related dementias are progressive and have significant effects on those diagnosed and their caregivers. Increasing emphasis on modifiable lifestyle factors to reduce dementia risk (e.g., physical activity, managing blood pressure, blood sugar, and weight) offers a sense of personal control and self-efficacy. Potential disadvantages include negative perceptions of individuals who do not modify their behaviors and labeling of dementia as a “lifestyle disease.” We examined whether behavior modification to reduce dementia risk affects attributions of responsibility and capability. In an online study, 242 participants (18-64 years, Mage=41.28, SDage=12.60) read a vignette describing ‘Patrick’s’ concerns about cognitive decline as reported to his physician. Although determined to be normal age-related change, the physician recommended exercise and diet changes to reduce Patrick’s dementia risk by improving cardiovascular health. Participants were randomly assigned to read about ‘Patrick,’ who either did or did not follow recommended lifestyle changes. Participants then rated Patrick’s responsibility for his current condition, capability for basic tasks, their willingness to socialize with him, and emotional reactions he provoked. Multivariate analysis of variance revealed that not modifying behavior led to rating Patrick as more responsible for his condition and less capable of decision-making compared to Patrick with behavior change; additionally, reading about Patrick without behavior modification resulted in lower willingness to socialize and greater levels of negative emotional reactions compared to Patrick with behavior modification (ps <.05). Findings highlight how behavior modification affects perceptions, suggesting the need for careful public health messaging about dementia risk and lifestyle modifications.

